# Rapidly progressive and fatal distant spontaneous gas gangrene due to *Clostridium septicum* after biopsy of malignant cecal mass

**DOI:** 10.1016/j.idcr.2021.e01129

**Published:** 2021-04-22

**Authors:** Geetha Sivasubramanian

**Affiliations:** University of California, San Francisco, 155 N Fresno St, Suite 307, Fresno, CA, 93701, United States

**Keywords:** Clostridium septicum, Spontaneous gas gangrene, Colon cancer

## Abstract

*Clostridium* species are known to cause myonecrosis and gas gangrene which are often fatal infections in the setting of trauma but also spontaneously in certain populations such as colorectal malignancy, immunosuppression, and neutropenia. We report a case of an 83-year-old male who developed fatal and rapidly progressive gas gangrene due to *Clostridium septicum* within 48 h after biopsy of suspected malignant cecal mass. To our knowledge, such a drastic, rapid and fatal presentation after a diagnostic biopsy of malignant mass has not been previously reported and is something to be watchful for in suspected colon cancer cases.

## Introduction

*Clostridium* species are known to cause gas gangrene or myonecrosis associated with high mortality and morbidity*. Clostridium perfringens* and *Clostridium septicum* are well known members of this family to cause these necrotizing infections. Whereas *Clostridium perfringens* often causes trauma associated, *Clostridium septicum* infections are usually spread hematogenously and can cause fatal spontaneous gas gangrene without associated trauma and have a unique association with colon cancer [1,2]. *Clostridium septicum* possesses several unique characteristics which enables it to cause this clinical syndrome. Unlike C. perfringens, it does not need strict anaerobic conditions to survive and hence is able to invade viable muscle tissue [[3], [4], [5]]. Also, the organism produces several toxins such as cytotoxic alpha, DNase beta, hyaluronidase gamma, hemolysin delta toxins, protease and neuraminidase toxins which enables tissue breakdown [4]. Additionally, it causes an exaggerated immune response which leads to multiorgan failure and shock [5].

## Case report

An 83-year-old Caucasian male with past medical history of hypertension and diabetes mellitus presented to the Emergency Department with a 10-day history of right sided abdominal pain which was constant and dull. He denied any fevers, chills, weight loss, nausea, vomiting, diarrhea, hematemesis, melena, or hematochezia. Physical exam showed normal vitals and normal exam without tenderness on palpation. His baseline labs were normal including normal white blood cell count, platelets, hemoglobin, liver function tests and creatinine. CT scan of the abdomen and pelvis showed eccentric mass-like thickening of the right side of the cecum with adjacent enlarged mesenteric and pelvic lymph nodes.

**Fig. 1:**
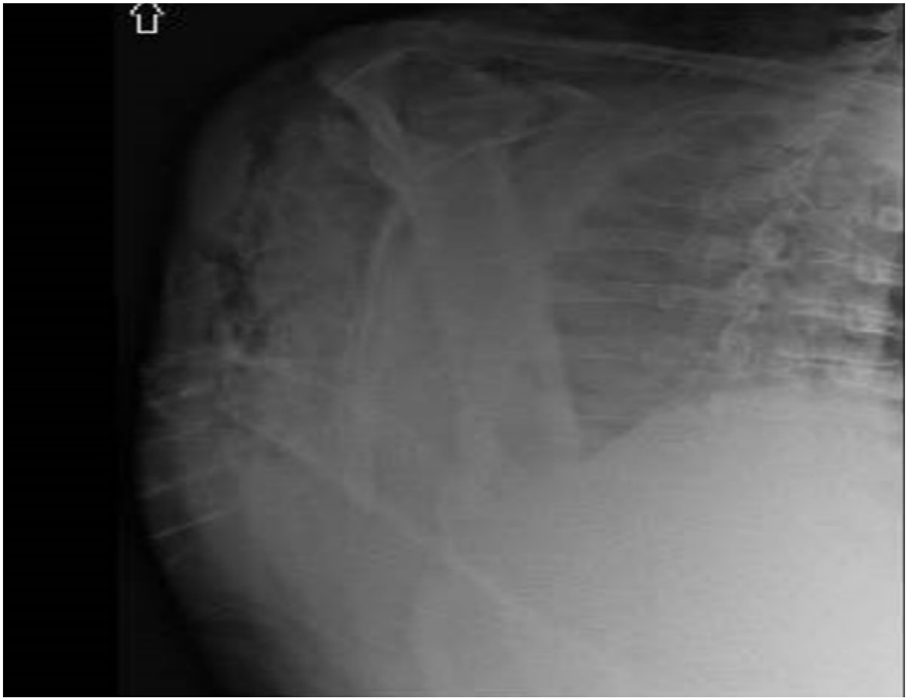
Right shoulder X Ray showing air in the soft tissues around shoulder.

**Fig. 2:**
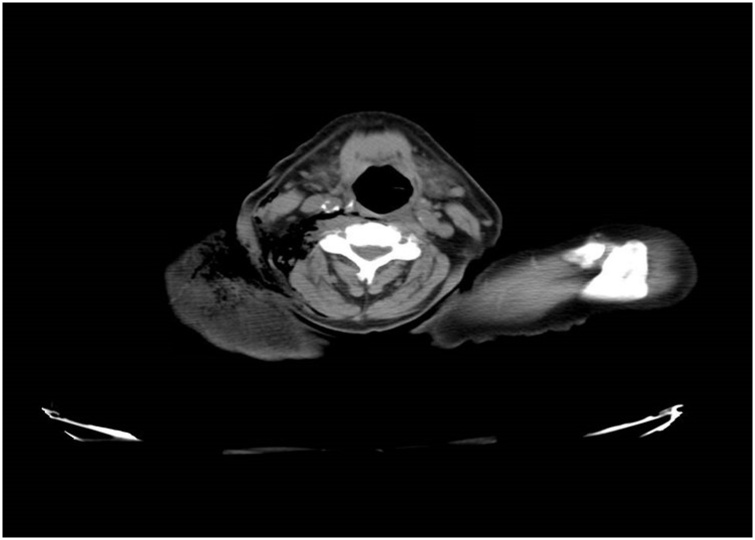
CT scan neck showing air in the soft tissues around right side of the neck.

**Fig. 3:**
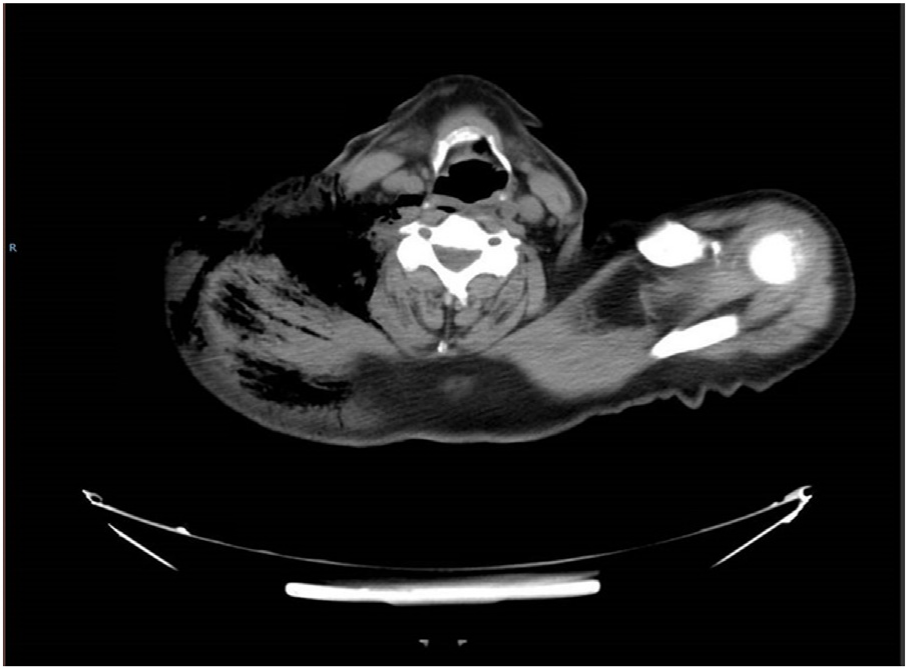
CT  chest showing air in the soft tissues right side of neck and chest wall.

**Fig. 4:**
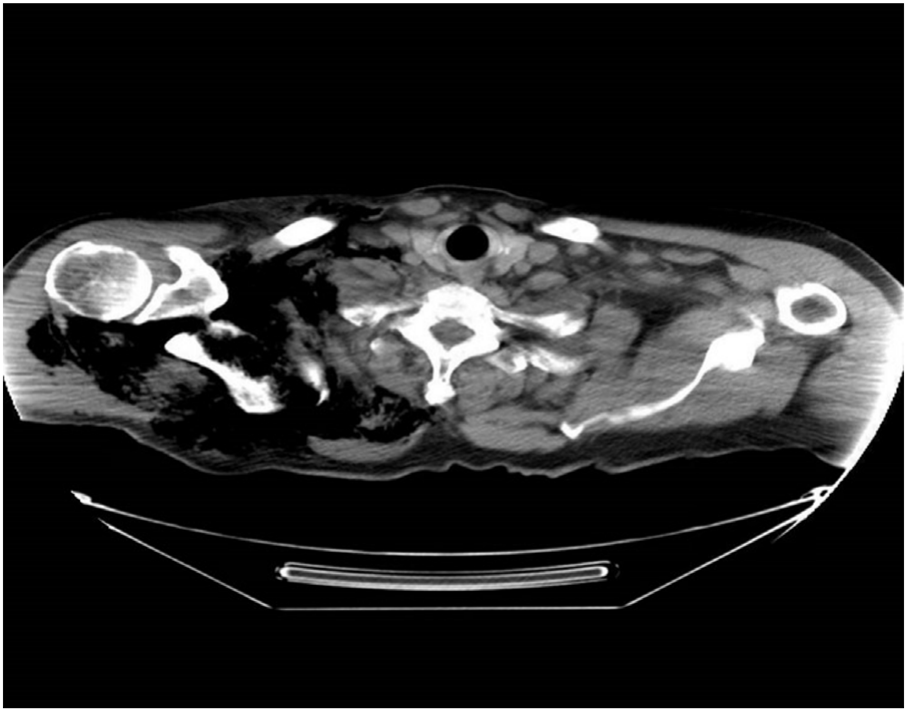
CT chest showing air in the soft tissues right side of neck and chest wall.

On hospital day 2, he underwent colonoscopy which showed a large fungating infiltrative mass of the cecum; multiple biopsies were taken from the mass. The next morning, on hospital day 3, he developed mild right shoulder pain and complained of being weak and fatigued along with decrease in urine output. Physical exam that morning showed preserved range of motion of right shoulder, no tenderness, or signs of inflammation but crepitus on palpation. Laboratory evaluation was remarkable for white blood cell count of 18,000 per/μL and creatinine of 2.83 mg/dL both of which were normal the previous day. Shoulder x-ray (Fig. 1) showed moderate sub-cutaneous emphysema over the right shoulder and neck. CT scan of neck and chest were done (Fig. 2, Fig. 3, Fig. 4), showed large amount of air within the subcutaneous tissues centered about the right neck, right thoracic inlet, shoulder, and chest wall without any pneumomediastinum or pneumothorax. CT of the abdomen was done pursuant to this and did not show any pneumoperitoneum or evidence of bowel perforation.

**Fig. 5:**
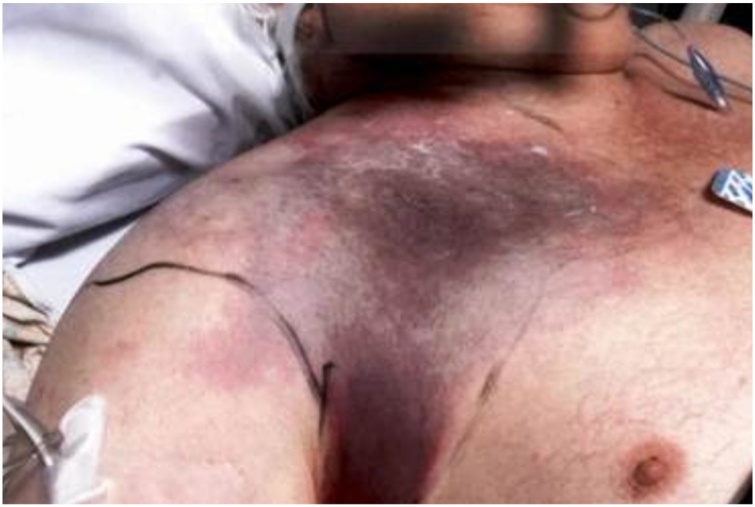
Discoloration of skin over right chest wall.

**Fig. 6:**
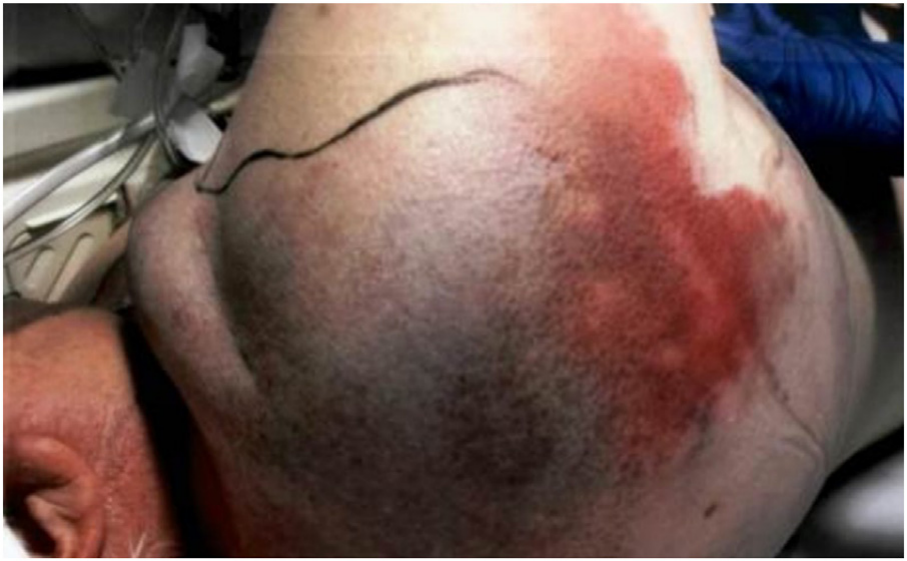
Discoloration of skin over right side of the back.

As the day progressed, he became progressively short of breath, hypotensive and started complaining of increasing pain in right shoulder and neck. He had to be transferred to the intensive care unit for hemodynamic support with vasopressors and mechanical ventilation. Broad spectrum intravenous vancomycin, meropenem and clindamycin were initiated. Physical exam later in the day started showing discoloration over the right shoulder, neck and extending into the back areas (Fig. 5, Fig. 6). He was on maximal vasopressor support by this time. After discussion, family opted for comfort measures only and the patient expired that night. His blood cultures later became positive for *Clostridium septicum* and pathology from biopsy of the cecal mass revealed a Grade 2 invasive adenocarcinoma with extensive acute inflammation. He was diagnosed with septic shock secondary to spontaneous gas gangrene of the right shoulder musculature due to *Clostridium septicum* which likely disseminated hematogenously from the cecal mass.

## Discussion

*Clostridium septicum* bacteremia is rapidly fatal, with survival rates close to 20 % in spontaneous gas gangrene [6,7]. Most of these cases are associated with colorectal malignancies [8]. The entry of *C. septicum* into the blood stream from the diseased colon is facilitated by inflammation in gut microenvironment as well as damage to the mucosa due to ulceration and necrosis [9,10]. Although most reported cases of such translocation are due to the disease itself, any further damage to the mucosal integrity such as an invasive biopsy that our patient had may lead to rapid translocation and bacteremia. The biopsy results from the patient’s mass did show significant associated inflammation which likely facilitated the bacteremia during instrumentation and biopsy.

Once *C. septicum* enters the blood stream, it can seed distant muscle tissue to cause myonecrosis. The early clinical features may be deceptive as in our patient’s case and may show only gas in soft tissue, subtle hemodynamic changes. The overt skin findings of tissue necrosis may not happen until multiorgan failure has set in [2]. Hence, having a high index of suspicion, especially in the setting of suspected colonic malignancy is required for diagnosis. Given the possibility of this complication after an invasive colonic biopsy, close monitoring and early intervention may be considered. Early, broad spectrum therapy with antimicrobials targeting clostridia such as penicillin and clindamycin is of paramount importance [3]. In addition to antimicrobials, diligent skin exam, early surgical consultation and debridement may be lifesaving [11].

## Conclusion

Although the association between *Clostridium septicum* and colon cancer is well known, distant spontaneous gas gangrene due to *C. septicum* bacteremia remains a diagnostic and therapeutic challenge due to early subtle presentation and rapidly progressive course. Instrumentation may exacerbate inflammation associated with diseased gut causing rapid translocation and distant myonecrosis. High index of suspicion, diligent monitoring and early intervention are crucial for managing this frequently fatal infection.

## Sources of funding for your research

None.

## Ethical approval

No concerns.

## Consent

No identifying patient information used.

## Author contribution

Written the article.

## Declaration of Competing Interest

The authors report no declarations of interest.

## References

[1] Srivastava I., Aldape M.J., Bryant A.E., Stevens D.L. (2017). Spontaneous C. septicum gas gangrene: a literature review. Anaerobe.

[2] Thompson K.M., Kruse B.T., Hedges M.A.S. (2018). Atraumatic clostridial myonecrosis in an Immunocompromised Host. J Emerg Med.

[3] Aldape M.J., Bayer C.R., Rice S.N., Bryant A.E., Stevens D.L. (2018). Comparative efficacy of antibiotics in treating experimental Clostridium septicum infection. Int J Antimicrob Agents.

[4] Stevens D.L., Aldape M.J., Bryant A.E. (2012). Life-threatening clostridial infections. Anaerobe.

[5] Dedemadi G., Sakellariou I., Kolinioti A., Lazaridis P., Anagnostou E. (2011). Clostridium septicum myonecrosis: a destructive and lethal condition. Am Surg.

[6] Zurmeyer S., Fotopoulou C., Braicu E., Schlichting U., Sehouli J. (2013). Clostridium septicum can cause distant myonecrosis in patients with ovarian cancer. Anticancer Res.

[7] Gray K.M., Padilla P.L., Sparks B., Dziewulski P. (2020). Distant myonecrosis by atraumatic Clostridium septicum infection in a patient with metastatic breast cancer. IDCases.

[8] Hermsen J.L., Schurr M.J., Kudsk K.A., Faucher L.D. (2008). Phenotyping Clostridium septicum infection: a surgeon’s infectious disease. J Surg Res.

[9] Kwong T.N.Y., Wang X., Nakatsu G., Chow T.C., Tipoe T., Dai R.Z.W. (2018). Association between bacteremia from specific microbes and subsequent diagnosis of colorectal cancer. Gastroenterology.

[10] Nagahama M., Takehara M., Rood J.I. (2018). Histotoxic clostridial infections. Microbiol Spectr.

[11] Corey E.C. (1991). Nontraumatic gas gangrene: case report and review of emergency therapeutics. J Emerg Med.

